# The association between dietary inflammatory index and diabetic foot ulcers in type 2 diabetes: a case-control study

**DOI:** 10.3389/fnut.2025.1683264

**Published:** 2025-10-15

**Authors:** Sultan Mohammed Alanazi, Ohoud Aied Alabonassir, Saleh Hussain A. Almasabi, Khalid Alhazmi, Majid Ali Alotni, Dahlia Soleman A. Mirdad, Salah Alghamdi

**Affiliations:** 1 Department of Medical Laboratory Technology, Northern Border University, Arar, Saudi Arabia; 2 Department of Medical Surgical Nursing, Nursing College, Najran University, Najran, Saudi Arabia; 3 Department of Clinical Laboratory Sciences, College of Applied Medical Sciences Najran University, Najran, Saudi Arabia; 4 Department of Pathology, Faculty of Medicine, University of Tabuk, Tabuk, Saudi Arabia; 5 Department of Medical Surgical, College of Nursing, Qassim University, Buraydah, Saudi Arabia; 6 Department of Basic Medical Sciences, College of Medicine, University of Jeddah, Jeddah, Saudi Arabia; 7 Department of Surgery, Faculty of Medicine, University of Tabuk, Tabuk, Saudi Arabia

**Keywords:** diabetic foot ulcers, type 2 diabetes mellitus, dietary inflammatory index, inflammation, case-control study

## Abstract

**Background:**

Diabetic foot ulcers (DFUs) are a major complication of type 2 diabetes mellitus (T2DM). This study examined the association between the Energy-adjusted Dietary Inflammatory Index (E-DII) and odds of DFUs.

**Methods:**

In a case–control study, 100 patients with DFUs and 150 T2DM controls were recruited. Dietary intake was assessed using a validated FFQ, and E-DII scores were calculated. Logistic regression estimated odds of DFUs across E-DII tertiles.

**Results:**

DFU patients had higher E-DII scores than controls (2.1 vs. 1.5, *p* < 0.001). E-DII scores were positively correlated with both ulcer duration (*ρ* = 0.32, *p* = 0.002) and PEDIS score (*ρ* = 0.45, *p* < 0.001). Multivariable logistic regression showed that higher E-DII scores were significantly associated with increased DFU risk (OR for highest tertile: 1.94 [1.02–3.67]).

**Conclusion:**

Our findings suggest that a pro-inflammatory diet, as measured by the E-DII, is associated with an increased odds and severity of DFUs in individuals with T2DM.

## Introduction

Diabetic foot ulcers (DFUs) are among the most debilitating complications of diabetes mellitus, contributing significantly to morbidity, healthcare costs, and diminished quality of life. These ulcers result from a complex interplay of peripheral neuropathy, peripheral arterial disease, and impaired wound healing, often exacerbated by chronic systemic inflammation (1). Emerging evidence underscores the pivotal role of inflammation in the pathogenesis and progression of DFUs, highlighting the need to explore modifiable factors that influence inflammatory status in diabetic individuals (2).

Dietary patterns have long been recognized as modulators of systemic inflammation. The Dietary Inflammatory Index (DII) is a validated tool that quantifies the inflammatory potential of a diet, with higher scores indicating a more pro-inflammatory dietary intake (3). Several studies have established associations between elevated DII scores and increased risk of chronic conditions such as cardiovascular diseases (4), certain cancers (5), and type 2 diabetes mellitus (T2DM) (6). For instance, a study utilizing data from the National Health and Nutrition Examination Survey found that each one-point increase in DII score was associated with a 13% higher odds of having diabetes, and among individuals with diabetes, higher DII scores correlated with greater disease severity, as indicated by elevated HbA1c levels (7).

Despite the established links between diet, systemic inflammation, and diabetes, there is a paucity of research specifically examining the relationship between dietary inflammatory potential and the occurrence of DFUs. While studies have explored the association between DII scores and some diabetic complications (7, 8), direct investigations into DII and DFUs remain limited. Therefore, the present case–control study aims to investigate the association between the Energy-adjusted Dietary Inflammatory Index (E-DII) scores and the risk of DFUs.

## Methods

### Study design and setting

This case–control study was conducted from January 1 to October 31, 2024 at Najran General Hospital, in Saudi Arabia. The study aimed to investigate the association between the E-DII and the odds of DFUs among individuals with T2DM. Ethical approval was obtained from the Institutional Review Board (IRB No. 2024-25E), and all participants provided written informed consent prior to enrollment. The study was conducted in accordance with the Declaration of Helsinki. Confidentiality and anonymity of participant data were maintained throughout the study.

### Participants

A total of 250 participants were recruited, comprising 100 cases and 150 controls. Cases were individuals aged 18 years or older with a confirmed diagnosis of T2DM and a current DFU, as diagnosed by a specialist based on clinical examination and relevant investigations. Controls were individuals aged 18 years or older with a confirmed diagnosis of T2DM but without any history or current presentation of DFUs. Inclusion criteria were confirmed diagnosis of T2DM for at least 1 year, presence of a DFU classified as Grade 1 or higher according to the Perfusion, Extent, Depth, Infection, and Sensation (PEDIS) classification system (9) and no history of DFUs for controls. Patients with the presence of other chronic inflammatory conditions (e.g., rheumatoid arthritis, inflammatory bowel disease), current use of anti-inflammatory medications or corticosteroids, and pregnancy or lactation were excluded.

### Data collection

Baseline data were collected through structured interviews. Dietary intake was assessed using a validated semi-quantitative Food Frequency Questionnaire (FFQ) comprising 147 food items, designed to capture habitual dietary intake over the past year. Participants reported the frequency and portion size of various foods and beverages consumed. DII was calculated based on the method developed by Shivappa et al., which involves scoring dietary components according to their inflammatory potential (3). Nutrient intake data were standardized and linked to a global reference database to compute individual DII scores, with higher scores indicating a more pro-inflammatory diet. In addition to the DII, the E-DII was also calculated to account for total energy intake. The E-DII provides a measure of the inflammatory potential of the diet independent of caloric intake, allowing for more accurate comparisons across individuals with varying energy consumption. The DII and E-DII calculations included the following food parameters: energy, protein, carbohydrate, total fat, saturated fatty acids, cholesterol, vitamin B12, and iron as the pro-inflammatory components; and thiamin, riboflavin, niacin, vitamin B6, folic acid, vitamin A, vitamin C, vitamin D, vitamin E, beta-carotene, magnesium, zinc, selenium, fiber, monounsaturated fatty acids, polyunsaturated fatty acids, n-3 fatty acids, n-6 fatty acids, garlic, onion, black tea, and caffeine as anti-inflammatory components (10). Intake of these components was standardized to global intake means and standard deviations derived from an international database. The standardized scores (Z-scores) were converted into centered proportions, which were then multiplied by the corresponding inflammatory effect scores for each food parameter (11). The products were summed to create an overall DII and E-DII score for each individual, reflecting the dietary inflammatory potential.

### Statistical analysis

All statistical analyses were conducted using SPSS software (version 25.0, IBM Corp., Armonk, NY). Continuous variables were expressed as means and standard deviations (SD), while categorical variables were presented as frequencies and percentages. The normality of continuous variables was evaluated using the Shapiro–Wilk test. To compare demographic, clinical, and dietary characteristics between participants with and without DFU, independent sample t-tests were used for continuous variables, and Chi-square tests were applied for categorical variables. Spearman’s rank correlation analysis was performed to assess the relationship between E-DII scores and ulcer characteristics (ulcer duration and PEDIS score) among individuals with DFU, given the non-parametric distribution of these variables. Participants were categorized into tertiles based on their E-DII scores to explore the association between dietary inflammatory potential and odds of DFU. Logistic regression analyses were used to calculate odds ratios (ORs) and 95% confidence intervals (CIs) for DFU occurrence across E-DII tertiles. Three regression models were developed: a crude model without adjustment; Model 1, adjusted for age and sex; and Model 2, adjusted for age, sex, education level, occupational status, hypertension status, smoking status, and BMI. A test for trend across tertiles was performed by including the median value of each tertile as a continuous variable in the regression models. A *p*-value less than 0.05 were considered statistically significant in all analyses.

## Results

### Baseline characteristics

The study included 250 individuals diagnosed with T2DM, with a mean age of 59 years. Of these, 100 were in the DFU group and 150 were in the non-DFU group. The DFU group had a higher mean age of 61.5 ± 7.38 years compared to 57.4 ± 8.69 years in the non-DFU group (*p* < 0.001). There was also a significant difference in education levels between the two groups (*p* = 0.03), with more participants in the DFU group having an education level below high school (Table 1).

**Table 1:** Sociodemographic characteristics of participants with and without diabetic foot ulcer.

Variable	DFU Group (*n* = 100)	Non-DFU Group (*n* = 150)	*p*-value
Age (years)	61.5 (7.38)	57.4 (8.69)	<0.001
Sex			
Female	42 (42)	88 (58.7)	
Male	58 (58)	42 (41.3)	0.007
Education level			
High school>	55 (55)	60 (40)	
High school	28 (28)	50 (33.3)	0.03
Diploma	10 (10)	25 (16.7)	
College	7 (7)	15 (10)	
Occupational status			
Yes	35 (35)	70 (46.7)	
No	65 (65)	80 (53.3)	0.069
Smoking status			
Never smoker	48 (48)	80 (53.3)	
Current smoker	38 (38)	54 (36)	
Former smoker	14 (14)	16 (10.7)	0.62

Data were expressed as mean (SD) and percent (%) for continuous and categorical variables, respectively. *p*-values were calculated based on independent sample *t*-test or Chi-square test. DFU, Diabetic foot ulcer.

The DFU group had a longer duration of diabetes (11.12 ± 6 years vs. 7.73 ± 5.43 years, *p* < 0.001) and higher levels of fasting blood sugar (174.76 ± 37.22 mg/dL vs. 154.92 ± 34.5 mg/dL, *p* < 0.001) and HbA1c (8.73 ± 1.06% vs. 7.58 ± 0.98%, *p* < 0.001). However, they had lower creatinine levels (70.87 ± 17.61 mg/dL vs. 78.76 ± 18.65 mg/dL, *p* = 0.001) and a higher prevalence of hypertension (64% vs. 50%, *p* = 0.037). Additionally, the DFU group showed significantly higher E-DII scores (2.1 ± 0.99) compared to the non-DFU group (1.51 ± 1.05, *p* < 0.001) (Table 2).

**Table 2:** Clinical and laboratory characteristics of participants.

Variable	DFU Group (*n* = 100)	Non-DFU Group (*n* = 150)	*p*-value
Diabetes duration, (years)	11.12 (6)	7.73 (5.43)	<0.001
FBS, (mg/dl)	174.76 (37.22)	154.92 (34.5)	<0.001
HbA1c (%)	8.73 (1.06)	7.58 (0.98)	<0.001
BMI (kg/m^2^)	28.07 (3.27)	28.23 (3.05)	0.68
cholesterol, (mg/dL)	196.07 (42.45)	192.05 (29.21)	0.37
creatinine, (mg/dL)	70.87 (17.61)	78.76 (18.65)	0.001
Hypertension	64 (64)	75 (50)	0.037
E-DII score	2.1 (0.99)	1.51 (1.05)	<0.001

Data were expressed as mean (SD) and percent (%) for continuous and categorical variables, respectively. *p-*values were calculated based on independent sample *t*-test or Chi-square test. DFU, Diabetic foot ulcer; FBS, fasting blood sugar; HbA1c, Hemoglobin A1c; BMI, Body mass index; E-DII, Energy-adjusted Dietary Inflammatory Index.

Characteristics of DFU in subjects based on size, duration, location, presence of deformity, Ankle-Brachial Index, and severity of DFU using the PEDIS assessment are presented in Table 3. Among those with DFU, the mean ulcer area was 4.16 ± 1.84 cm^2^, with average ulcer duration of 8.31 ± 3.36 weeks. Most ulcers were located on the forefoot (60%), followed by the midfoot (25%) and heel (15%). The mean PEDIS score was 3.48 ± 0.8, and neuropathy was present in 81% of participants. The mean ankle-brachial index was 0.84 ± 0.15, and foot deformities were noted in 41% of patients.

**Table 3:** Diabetic foot ulcer characteristics in DFU group only.

Variable	DFU Group (*n* = 100)
Ulcer area (cm^2^)	4.16 (1.84)
Ulcer duration (weeks)	8.31 (3.36)
Ulcer location	Forefoot: 60 (60%), Midfoot: 25 (25%), Heel: 15 (15%)
PEDIS score	3.48 (0.8)
Neuropathy present	81 (81)
Ankle-Brachial Index	0.84 (0.15)
Deformity present	41 (41)

Data were expressed as mean (SD) and percent (%) for continuous and categorical variables, respectively. DFU, Diabetic foot ulcer; PEDIS score, perfusion, extend, depth, infection, sensation score.

### Association between E-DII and DFU

E-DII scores were significantly and positively correlated with both ulcer duration (*ρ* = 0.32, *p* = 0.002) and PEDIS score (*ρ* = 0.45, *p* < 0.001), indicating that more pro-inflammatory diets were associated with more severe and prolonged ulcers (Table 4).

**Table 4:** Correlation between E-DII and ulcer characteristics among DFU patients.

Ulcer characteristic	Spearman’s ρ	*p*-value
Ulcer duration (weeks)	0.32	0.002
PEDIS score	0.45	<0.001

In multivariable logistic regression models, higher E-DII scores were positively associated with DFU. Compared to the lowest tertile (T1), participants in the highest tertile (T3) had significantly higher odds of DFU in the crude model (OR = 2.05, 95% CI: 1.12–3.76), in Model 1 adjusted for age and sex (OR = 2.09, 95% CI: 1.12–3.91), and in Model 2 further adjusted for education level, hypertension status, smoking, and BMI (OR = 1.94, 95% CI: 1.02–3.67), whereas the middle tertile (T2) did not show a significant association. When E-DII was modeled as a continuous variable, each one-unit increase in E-DII score was associated with a modest increase in odds of DFU in the crude model (OR = 1.16, 95% CI: 1.01–1.32) and similar results were observed in Model 1 (OR = 1.16, 95% CI: 1.00–1.33) (Table 5).

**Table 5:** Crude and adjusted associations between E-DII Score and DFU among the study population.

Characteristic	DFU/Control	Crude OR (95% CI)	Model 1 OR (95% CI)	Model 2 OR (95% CI)
E-DII (continuous)	-	1.16 (1.01–1.32)	1.16 (1–1.33)	1.15 (0.99–1.33)
E-DII categorical				
T1	32/60	1 (ref)	1 (ref)	1 (ref)
T2	49/23	1.13 (0.59–2.18)	1 (0.5–1.98)	1.03 (0.51–2.1)
T3	41/45	2.05 (1.12–3.76)	2.09 (1.12–3.91)	1.94 (1.02–3.67)
*p*-trend	-	0.019	0.021	0.044

Model 1 was adjusted for age and sex and Model 2 was adjusted for: age, sex, education status, Occupational status, Hypertension status, smoking status, and body mass index. DFU, Diabetic foot ulcer; EDII, Energy adjusted dietary inflammatory index, OR, Odds Ratio, CI, Confidence Interval.

## Discussion

This study investigated the association between the E-DII and the odds and severity of DFUs in individuals with T2DM. The findings revealed that patients with DFUs had significantly higher E-DII scores compared to those without DFUs, indicating a more pro-inflammatory diet. Furthermore, higher E-DII scores were positively correlated with longer ulcer duration and higher PEDIS scores, suggesting that a pro-inflammatory diet may contribute to both the development and severity of DFUs. Multivariable logistic regression analyses demonstrated that individuals in the highest E-DII tertile had nearly double the odds of developing DFUs compared to those in the lowest tertile, even after adjusting for potential confounders such as age, sex, education level, hypertension status, smoking, and BMI. These results underscore the potential role of dietary inflammation in the pathogenesis of DFUs and highlight the importance of dietary interventions in the management of patients with T2DM.

These results align with previous research highlighting the role of dietary inflammation in diabetes-related complications. For instance, a meta-analysis encompassing 48 studies with over 1.6 million participants found that higher DII scores were associated with an increased risk of T2DM, particularly in high-quality studies (12). Similarly, another meta-analysis reported a 32% higher risk of diabetes mellitus among individuals consuming pro-inflammatory diets (6). Furthermore, a case–control study observed that individuals with higher DII scores had increased odds of developing diabetic sensorimotor polyneuropathy, emphasizing the impact of pro-inflammatory diets on diabetes complications (8). Another study examined the relationship between DII and diabetic kidney disease (DKD). The findings indicated that elevated DII levels were significantly associated with an increased risk of DKD, with a notable non-linear correlation observed between DII scores and DKD risk (13). To our knowledge, this is the first study to examine the association between dietary inflammatory potential and diabetic foot ulcers. While prior work has shown that inflammatory diets worsen neuropathy and nephropathy in diabetes (8, 13). Unlike prior work that focused on metabolic control or microvascular complications, our findings extend the role of the E-DII to a complication characterized by impaired wound healing and high clinical burden. By showing that higher E-DII scores are not only associated with the presence of DFUs but also with ulcer severity and duration, this study highlights diet as a potentially modifiable factor in both the prevention and management of DFUs. In addition to the association between DII and the odds of diabetes and its related complications mentioned above, the DII has also been associated with prediabetes and insulin resistance. A study investigating American adults found that higher DII scores correlated with increased fasting plasma glucose, fasting serum insulin, and homeostatic model assessment of insulin resistance (HOMA-IR) scores, indicating a greater odds of prediabetes and insulin resistance (14). These findings suggest that pro-inflammatory diets may contribute to the early stages of glucose metabolism disorders.

The observed association between higher E-DII scores and increased DFU risk may be explained by several molecular mechanisms. Pro-inflammatory diets are known to elevate systemic levels of inflammatory cytokines such as interleukin-6 (IL-6) and tumor necrosis factor-alpha (TNF-*α*) (15), which can impair endothelial function and reduce blood flow to peripheral tissues, exacerbating the risk of ulceration (16). Additionally, chronic inflammation can lead to increased oxidative stress, further damaging vascular structures and impairing wound healing processes (17). Elevated levels of C-reactive protein (CRP), a marker of systemic inflammation, have also been associated with delayed wound healing in diabetic patients (18). Moreover, anti-inflammatory diets may influence the expression of matrix metalloproteinases (MMPs), enzymes that degrade extracellular matrix components and are involved in tissue remodeling during wound healing (19). An imbalance in MMP activity can disrupt the normal healing process, leading to chronic, non-healing ulcers (20).

Our study has several strengths, including a well-characterized cohort of individuals with T2DM and the use of the E-DII, a validated measure of dietary inflammation. The comprehensive assessment of DFU characteristics, including ulcer size, duration, location, and PEDIS scores, allowed for a detailed analysis of the relationship between dietary inflammation and ulcer severity. However, several limitations should be considered. First, dietary intake relied on self-reported data from a semi-quantitative FFQ, which may be subject to recall bias and could affect the accuracy of E-DII estimation. Although we used a validated FFQ and trained interviewers to reduce this bias, inaccuracies remain possible, particularly in patients with diabetes who may underreport unhealthy food consumption. Such non-differential misclassification could attenuate the observed associations between the E-DII and diabetic foot ulcers. Future research should consider combining FFQs with more objective measures, such as multiple 24-h dietary recalls or nutritional biomarkers, to provide a more accurate assessment of dietary inflammatory potential. Second, potential measurement errors in dietary assessment were not formally assessed through sensitivity analyses, such as using alternative scoring methods or repeated dietary measurements, which may influence the observed associations. Third, the relatively small sample size, particularly after grouping participants into E-DII tertiles, may have limited the statistical power to detect smaller effect sizes. Additionally, the cross-sectional design precludes conclusions about causality, the study population was drawn from a single center, and residual confounding by unmeasured factors, such as physical activity levels or socioeconomic status, cannot be ruled out.

Despite these limitations, our study provides valuable insights into the potential role of dietary inflammation in DFU pathogenesis. Future research should focus on longitudinal studies to establish temporal relationships between dietary inflammation and DFU development. Interventional studies assessing the impact of anti-inflammatory dietary patterns on DFU incidence and healing outcomes would be particularly informative. Mechanistic studies exploring the molecular pathways linking dietary components to inflammation and wound healing processes in diabetic patients are warranted. Expanding the study population to include diverse demographic and geographic groups would enhance the generalizability of the findings. Ultimately, integrating dietary assessments and interventions into standard care for patients with T2DM may offer a promising avenue for reducing the burden of DFUs.

## Conclusion

Our findings show that higher E-DII scores were not only more prevalent among patients with DFUs but also correlated with prolonged ulcer duration and greater PEDIS scores, suggesting more severe clinical manifestations. These results underscore the critical role that diet-induced inflammation may play in the pathophysiology and progression of diabetic complications, particularly those related to impaired wound healing and vascular function in the lower extremities.

## Data Availability

The original contributions presented in the study are included in the article/supplementary material, further inquiries can be directed to the corresponding author.
